# Crystalline Ultrastructures, Inflammatory Elements, and Neoangiogenesis Are Present in Inconspicuous Aortic Valve Tissue

**DOI:** 10.4061/2010/685926

**Published:** 2010-12-28

**Authors:** P. Dorfmüller, D. Bazin, S. Aubert, R. Weil, F. Brisset, M. Daudon, F. Capron, I. Brochériou

**Affiliations:** ^1^Service d'Anatomie et de Cytologie Pathologiques, Hôpital de la Pitié-Salpêtrière, 47-80 Boulevard de l'Hôpital, Assistance Publique-Hôpitaux de Paris, Université Pierre et Marie Curie, 75013 Paris, France; ^2^Service d'Anatomie et de Cytologie Pathologiques, Hôpital Marie Lannelongue, Université Paris-Sud, 133 Avenue de la Résistance, 92350 Le Plessis Robinson, France; ^3^Laboratoire de Physique des Solides, UMR 8502, Université Paris-Sud Orsay, Bâtiment 510, 91405 Orsay, France; ^4^Service de Chirurgie Cardiaque, Hôpital de la Pitié-Salpêtrière, 47-80 Boulevard de l'Hôpital, Assistance Publique-Hôpitaux de Paris, Université Pierre et Marie Curie, 75013 Paris, France

## Abstract

Morbidity from calcific aortic valve disease (CAVD) is increasing. Recent studies suggest early reversible changes involving inflammation and neoangiogenesis. We hypothesized that microcalcifications, chemokines, and growth factors are present in unaffected regions of calcific aortic valves. We studied aortic valves from 4 patients with CAVD and from 1 control, using immunohistochemistry, scanning electron microscopy, and infrared spectrography. We revealed clusters of capillary neovessels in calcified (ECC), to a lesser extent in noncalcified (ECN) areas. Endothelial cells proved constant expression of SDF-1 in ECC, ECN, and endothelial cells from valvular surface (ECS). Its receptor CXCR4 was expressed in ECC. IL-6 expression correlated with CXCR4 staining and presence of lymphocytes. VEGF was expressed by ECS, its receptor by ECC and ECN. Crystalline ultrastructures were found on the surface of histologically noncalcified areas (HNCAs), spectrography revealed calcium hydroxylapatite. Our results demonstrate that crystalline ultrastructures are present in HNCAs, undergoing neoangiogenesis in an inflammatory context. These alterations could be an early witness of disease and an opening to therapy.

## 1. Introduction

Calcific aortic valve disease (CAVD) is the most common valvular lesion in Western countries and the major cause of aortic valve replacement. It is associated with a higher death risk from cardiovascular causes which is increased up to 50 per cent, even in the absence of a hemodynamically significant obstruction [1, 2]. With an aging population, CAVD is becoming an important public health problem. Hitherto, a cure for symptomatic calcific aortic valve disease relies entirely within surgical procedures with valve replacement. Until recently, the concept that CAVD is a degenerative and unmodifiable process basically induced by long-lasting mechanical stress was generally accepted [3, 4]. Interestingly recent studies suggest that valvular changes might not be an unmodifiable degenerative disease but rather a dynamic process involving inflammation, lipid infiltration, dystrophic calcification, and endothelial dysfunction [5–7]. 

Chemokines and growth factors play an important role in several pathophysiological processes such as inflammation and immunity. Chemokine receptor CXCR4 is expressed on monocytes, B-lymphocytes, and most T cells [8–10]. It has been determined that CXCR4, which is present on many different types of cells, is activated by only one ligand, stromal derived factor 1 (SDF-1) and mediates several different activities such as chemotaxis, adhesion, proliferation, survival, and, in some cells, apoptosis [11–13]. Activation of CXCR4 on lymphocytes and monocytes stimulates chemotaxis, resulting in recruitment to sites of immune and inflammatory reactions. Hematopoietic and endothelial progenitor cells express CXCR4, and release of SDF-1 by bone marrow stromal cells mediates sequestration and homing of these progenitor cells to the bone marrow [14, 15]. Vice versa, SDF-1 has also been implicated in revascularization of ischemic hind limbs through recruitment of CXCR4+ hemangiocytes [16, 17], and, recently, SDF-1 and its receptor CXCR4 have been shown to support development of vascular blood supply within inflammatory atherosclerotic plaques [18]. On the other hand some vascular growth factors, in particular VEGF and its receptors (VEGFR1 and 2), are known to be involved in the evolution of malignancies, intraocular neovascular disorders, and atherosclerotic plaque formation [19]. Pathological neoangiogenesis—whether in the context of inflammation or cancer—is partly put onto the account of VEGF/VEGFR signalling [20]. We hypothesized that SDF-1 and VEGF might act as regulators of angiogenesis within primarily concerned valves, depending on their expression and presence of their corresponding receptors on endothelial cells.

Due to routine experience with minimal valvular alterations including discrete calcification and associated capillary-like neovessels, we have been interested in possible early stage changes of clinically silent and macroscopically inconspicuous valve areas/valves. We therefore hypothesized that early fibrous remodeling and neoangiogenesis within aortic valves could be initiated by ultrasctructural calcific alterations.

## 2. Methods

Tissue samples corresponding to aortic valve resections were collected from patients undergoing surgical treatment for CAVD (*n* = 4) and aortic insufficiency due to aortic arch aneurysms (*n* = 1) in the Department of Cardiac Surgery (Hôpital de la Pitié-Salpêtrière, Paris). After resection, specimens were fixed in formalin and dissected. Half of the specimens were processed to paraffin blocks and serially sectioned at 3 *μ*m thickness. Slides were stained with haematoxylin, eosin, and saffron. The other half was discriminated into calcified and noncalcified areas and eventually inserted into X-ray permeable kapton film for submission to X-ray fluorescence and X-ray diffraction experiments. 

Each sample was investigated by FT-IR spectroscopy using a Fourier transform infrared spectrometer Bruker IFS25 (Bruker Spectrospin, Wissembourg, France) between 4000 and 400 cm^−1^ with a resolution of 4 cm^−1^ [21]. A Zeiss SUPRA55-VP-type scanning electron microscope (SEM) was used for microstructure observation. To maintain integrity of the aortic valve, measurements were performed at low voltage (1.4 KV) and without the usual deposits of carbon at the surface of the sample. Immunohistochemical analyses were performed on formalin-fixed samples. Paraffin-embedded sections were stained with monoclonal mouse antihuman primary antibodies against von Willebrand factor (Dako reference A0082; dilution, 1 : 200), CD3 (Dako reference F7.2.38; dilution, 1 : 100), CXCL12/SDF-1 (RD system; clone 79018, dilution 1/30), CXCR4 (RD system; clone 12G5, dilution 1/100) and polyclonal rabbit antihuman antibodies against CD117 (Dako reference K0678; dilution, 1 : 200), IL-6 (Abcam reference ab6672; dilution 1 : 400). According to the manufacturer's recommendations, DAKO LSAB kit k0673 was used for primary antibody detection and Fast Red TR substrate system was the chromogen. We assessed immunohistochemical staining of endothelial cells (ECs) in three anatomical compartments: EC of the leaflet surface (ECS), EC of neovessels near calcified areas (ECC), and EC of neovessels distant to calcified areas (ECN).

The study was approved and executed in compliance with the institutional review board of the Pitié-Salpêtrière Hospital and all subjects gave informed consent concerning the use of tissue.

## 3. Results

### 3.1. Histopathology

**Figure 1:**
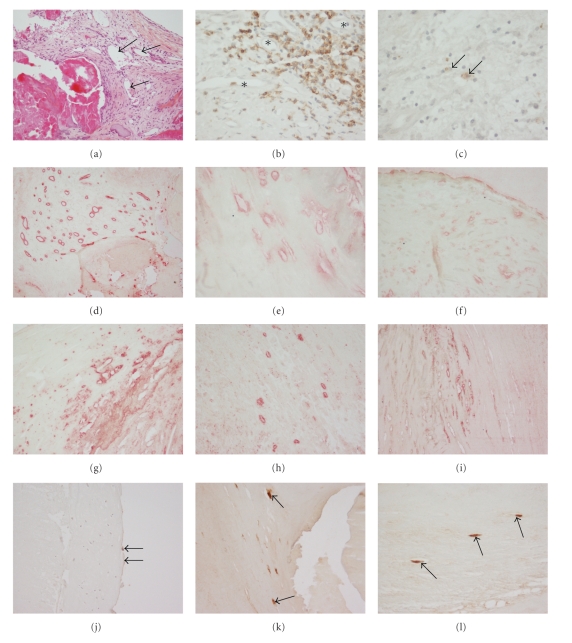
Immunohistochemical pattern of inflammatory infiltrate and neovascular endothelial cells in microscopically calcified (ECC) and noncalcified (ECN) areas, as well as of surface endothelial cells (ECS) in aortic valves from patients with calcific aortic stenosis. (a) Hematoxylin-Eosin-Saffron staining, note multiple capillary neovessels near calcified area (arrows); (b) CD3+ lymphocytes in the range of neovessels (asterisks) near calcific lesions; (c) CD117+ cells corresponding to mast cells are sparsely present. (d) Neovessels of all subgroups are highlightened by FVIII staining (here: ECC) (e) CXCL12 (ECC); (f) CXCL12 (ECN); (g) CXCR4 (ECC); (h) CXCR4 (ECN); (i) IL-6 (ECC); (j) VEGF in surface endothelial cells (arrows on ECS); (k) VEGFR (arrows on ECC); (l) VEGFR (arrows on ECN). Images (a),(d),(f),(g),(h),(i),(j), and (k): magnification ×100; images (b),(c),(e), and (l): magnification ×200.

The morphologic hallmarks of CAVD were heaped-up calcified masses within the aortic cusps that protruded into the outflow surfaces. Calcific deposits distorted the cuspal architecture, primarily at the bases. The free cuspal edges were usually not involved. On the leaflet profile calcifications were located within the valvular fibrosa, at the point of maximal cusp flexion. Cusps were heavily fibrosed and frequently presented chronic inflammatory infiltrate mainly consisting of lymphocytes, macrophages, and masts cells. Sporadically, foreign body granulomas with giant cells were present near calcifications. Numerous blood microvessels within calcified areas were observed in close association to inflammatory infiltrates and frequently disposed in a nest-like pattern (Figure 1(a)). Microvessels revealed different phenotypes with thin-walled capillary-like appearance or thickened arteriolar wall structure. Intra- or extracellular lipid depositions, as seen in arterial atheromatous lesions, were not observed. The histological valve architecture distant to calcified areas was preserved. Histomorphological correlates to calcification or microcalcification at this site were not observed. However, cusps showed some fibrous remodelling, and few thin-walled capillary neovessels were present. The control sample (Bentall surgery) grossly corresponded to a normal valve leaflet anatomy with a single layer of endothelial cells lining the spongiosa layer on the aortic side with loosely organized connective tissue and an elastin-rich ventricularis layer on the ventricular side. Although aortic valves are considered as nonvascularized tissues, few capillary neovessels, but no inflammatory infiltrates, were present on the control sample. Calcifications or major fibrous remodeling were not observed.

### 3.2. Immunohistochemistry

EC from all samples and from all compartments expressed vWF and CXCL12. CXCR4 expression was observed in ECC, while ECN and ECS were sporadically stained or completely negative. IL-6 was expressed in ECC and ECS, while mostly negative in ECN. The control sample presented with a different expression profile: as by definition, only ECN and ECS were present; while both EC subgroups were easily detected by vWF expression and stained for CXCL12, ECN were negative for IL-6, while ECS moderately stained.

VEGF was constantly expressed in ECS of all valves and, to a lesser extent, in ECC. VEGFR1 was expressed in both ECC and ECN, while ECS was negative. ECN in the control sample revealed expression of VEGFR1.

Inflammatory infiltrate in the range of neovessels mainly revealed CD3+ lymphocytes and CD117+ mast cells. In an attempt to differentiate mast-cells from CD117+ bone marrow-derived progenitor cells, slides were secondarily counter stained with Toluidine-Blue; CD117-positive/Toluidine-negative cells were not observed. Immunohistochemical results are presented in Figures 1(b)–1(l).

### 3.3. Scanning Electron Microscopy

**Figure 2:**
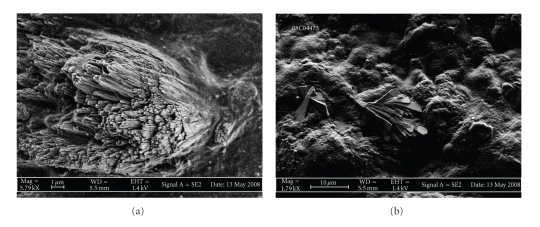
Scanning electron micrographs of crystalline ultrastructures on the surface of inconspicuous valve areas in patients suffering from CAVD. (a) Bundles of faceted microrods are inserted into the leaflet surface. Magnification ×5790. (b) Surface areas are covered with round-shaped or sphere-like, flat structures adherent to the leaflet surface. Note the additional small bundle of plate-like facetted microrods (center). Magnification ×1790.

Scanning Electron Microscopy (SEM) analysis of the leaflet surface within noncalcified areas revealed the presence of two types of three-dimensional crystal structures: we differentiated plate-like, facetted microrods which were vertically inserted into the endothelial surface, measuring approximately 10 *μ*m. These heterogeneously distributed structures where associated with 5–8 *μ*m large sphere- or nodule-like structures, focally covering the leaflet surface. The same ultrasctructural modifications were—to a lesser extent—present in the control sample (Figures 2(a) and 2(b)). In contrast, calcified areas massively displayed large crystalline accumulations of 100 *μ*m and more, extensively covering the analyzed valve areas.

### 3.4. EDX Analysis

**Figure 3:**
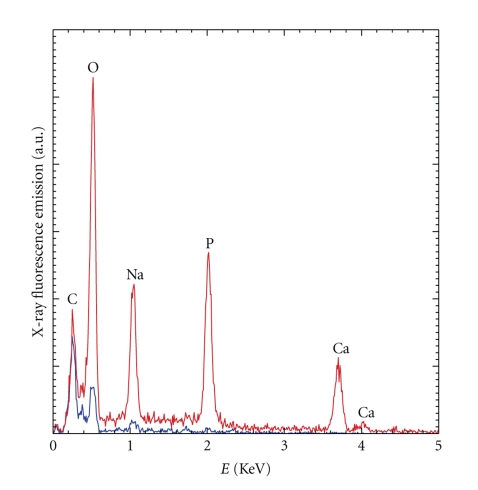
EDX spectra collected for the ultrastructures presented above (blue graph) and for massively calcified lesions within the same valve (red graph) (SEM not shown). The contributions of light elements such as C (carbon), O (oxygen), and Na (sodium) are predominating within crystalline ultrastructures, while massive calcifications display higher peaks of elements such as P (phosphor) and Ca (calcium). Results are fitting into the chemical composition given by FT-IR (see beneath).

X-ray fluorescence emission revealed a significant phosphor peak in calcified and a small elevation of phosphor in noncalcified areas. No calcium peak was noticeable for noncalcified areas, whereas present in calcified regions. Light elements, such as carbonate, oxygen, and sodium were present in both regions (Figure 3).

### 3.5. FT-IR Spectroscopy

Calcium apatite and amorphous carbonated calcium phosphate were the major components in grossly calcified areas. Inconspicuous areas with crystalline ultrastructures only contained calcium apatite. Approximately 80% of the material in grossly calcified areas corresponded to calcium apatite, and 10% of calcium apatite was found in grossly noncalcified areas. Calcium apatite was also present in the control sample (Bentall surgery). Higher amounts of triglycerides were retrieved within inconspicuous valve areas, as compared to calcific lesions (Table 1).

## 4. Discussion

In order to detect ultrastructural pathological transformations in valve structure, we analyzed noncalcified and calcified areas of human aortic valves which had been surgically removed due to symptomatic calcific stenosis, as well as one totally inconspicuous valve excised in the context of Bentall surgery in a patient with nonatherosclerotic aortic aneurysm. After histological study of the samples, we submitted them to scanning electron microscopic and Fourier transform—infrared spectroscopy in an attempt to eventually define an elementary profile indicating an initial change in structural organization, possibly preceding manifest remodeling.

Our scanning electron micrographs suggest that crystalline ultrastructures of different appearance are present on the surface of histologically inconspicuous valve tissue. SEM analysis allowed us to differentiate two types of crystalline ultrastructures, within noncalcified areas: we discriminated smaller, spherical, or nodule-like structures partly covering the surface and accumulations of larger, plate-like facetted microrods radially inserted into the valve leaflet. Our observations match with the ultrastructural aspect of typical biological apatites within calcified lesions of human aortic valves: a physochemical and ultrastructural study by Mikroulis and coworkers of native aortic valves and porcine aortic bioprostheses revealed two types of coexisting valve deposits, that is, large (>20 micrometers) and medium (5–20 micrometers) plate-like crystals as well as microcrystalline (<5 micrometers) calcium phosphate mineral formations [22]. Their results suggest that the mineral salt of calcified valves is a mixture of calcium phosphate phases, such as dicalcium phosphate dihydrate (DCPD), octacalcium phosphate (OCP), and hydroxyapatite (HAP). DCPD and OCP are suggested to be precursor phases transformed to HAP by hydrolysis. Of importance, the authors found a lower value of the Ca/P molar ratio in the bioprostheses as compared with native valves. This difference was ascribed to a higher content of precursor phases, such as DCPD and OCP in bioprosthetic valves, subsequently transformed into HAP. In terms of ultrastructural morphology and chemical composition we have found resembling pattern within macroscopically inconspicuous valve areas. Noteworthy, Ca/P ratio was significantly lower in macroscopically noncalcified areas, as compared to grossly calcified lesions. FT-IR results for the calcified samples showed that calcium apatite and amorphous carbonated calcium phosphate are the major components in grossly calcified areas. In contrast, inconspicuous areas with crystalline ultrastructures only contain calcium apatite, even though in different proportions: while approximately 80% of the material in grossly calcified areas is made of calcium apatite, 10% of calcium apatite is found in inconspicuous areas. Of note, calcium apatite is also present in the control sample (Bentall surgery). In addition, we observed at least comparable, if not slightly higher, amounts of triglycerides within inconspicuous valve areas, as compared with calcific lesions. It is of note that several studies dedicated to the morphology of triglyceride crystals [23, 24] indicate that these ultrastructures present a sphere-like or a layered pattern, similar to our leaflet covering micronodules. Finally, our EDX spectra reveal the presence of sodium in calcified and in inconspicuous areas, possibly signifying additional involvement of sodium urate crystals. As expected, analysis of grossly calcified aortic valve areas showed important ultrastructural modifications with large crystalline deposits of calciumapatite associated with fibrous remodeling. However, histological and ultrastructural morphology appears to be discordant in inconspicuous, grossly noncalcified valve areas. As stated above, our SEM analysis revealed presence of facetted crystalline microrods and layered micronodules on the leaflet surface. These structures morphologically displayed tight adherence to the endothelial surface and might in terms of flow dynamics represent a critical source of mechanical and/or oxidative stress through flow turbulences. In a recent study, Miller and coworkers have reported significantly increased oxidative stress and altered antioxidant mechanisms in stenotic aortic heart valves, as compared to normal heart valves [25]. Interestingly, they have differentiated calcified from noncalcified regions of stenotic valves and found significant dysregulation of antioxidant enzymes superoxide dismutase (SOD) 1 and 3 in noncalcified areas, suggesting that an impairment of SOD upregulating mechanisms could contribute in a tissue damaging increase of superoxide levels. Of note, endothelial injury is considered as an early event in the formation of a pathological calcification especially in the spongiosa layer on the aortic side of the valve [26–28]. Further, previous studies have reported endothelial cell dysfunction in apparently calcified aortic valves with deregulation of endothelial cell markers such as ICAM-1, VCAM-1, IL-6, and FVIII, suggesting that endothelial cell dysfunction might to some extent be present in early stages of disease evolution [29–32]. Interestingly, Ewence and coworkers report a potential greater risk for calcium phosphate micro- and nanoparticles in early stages of atherosclerosis as compared to larger, radiologically visible deposits in more advanced lesions through an effective induction of various inflammatory pathways [33] and it may be speculated that these inflammatory mediators are expressed in the presence of valvular submicroscopic calcifications. However, our immunohistochemical experiments revealed different EC phenotypes, depending on the valve area and its pathological remodeling: we observed constant SDF-1 expression in EC from every compartment in human calcified aortic valves. In contrast, its receptor CXCR4 was mainly expressed in microvessel accumulations near calcified nodules and to a lesser extent in leaflet lining endothelium. Staining was inversely correlating with the distance to calcified areas. Interestingly a similar pattern was observed in the expression of the proinflammatory cytokine IL-6, with prominent staining in EC near grossly lesioned valve areas. It has been speculated that augmentation of CXCR4 signalling in blood vessels near calcifications induces proliferation of neovessels and promotes inflammatory cell recruitment from blood [13–17]. The interplay of chronic inflammation and tissue calcification has many similarities with atherosclerosis of systemic arteries: the appearance and role of neoangiogenesis in calcified atheromas have been largely described and are still controversially discussed by several groups [34–36]. The formation of neovessels within a primary inflammatory lesion is considered as an early event in CAVD and might contribute to lesion progression by direct homing of inflammatory cells towards a chemokine gradient [18, 37]. The pathomechanisms of endothelial cell recruitment and neovessel organization within primary lesions remain to be clarified. Some investigators have detected significant numbers of endothelial progenitor cells predominantly localized within the valvular fibrosa of calcified valves, suggesting that cells of extravalvular origin contribute to aortic valve degeneration [38]. Others have suggested that recruitment of circulating progenitor cells at the site of injured tissues facilitates neovascularization through the VEGF/SDF-1 system, both potent angiogenic factors [39]. In fact, it has been shown that VEGF expression is increased in grossly calcified aortic valve tissue and associated with neovascularization, as well as with expression of bone substrate proteins, such as osteopontin and osteocalcin [33]. Our results confirm that VEGF is mainly expressed in endothelial cells of the leaflet surface. Importantly, we found that its receptor VEGFR1 is expressed in EC of neovessels near calcified areas, as well as in noncalcified areas, suggesting an ongoing angiogenetic loop within macroscopically bland areas with ultrastructural crystalline deposits. Regarding the possible presence of progenitor cells, our immunohistochemical experiments identified only granulated CD117-positive cells being toluidine-positive, as well, and hence most probably corresponding to masts cells and not to progenitor cells. Nevertheless, manifest calcific valve remodeling could be initiated after a primary ultrasctructural event, through triggering of inflammatory cascades orchestrated by cytokines, such as IL-6 and subsequent pathologic angiogenesis via paracrine activity of VEGF/SDF-1, finally rendering the lesion site “active.”

The mechanism of CAVD remains largely unknown compared to our understanding of the mechanisms underlying atherosclerotic diseases. We hypothesize that the SDF-1/CXCR4 and the VEGF/VEGFR system could have a pivotal role in the initiation and development of CAVD. Our observations demonstrate that expression of SDF-1 and its receptor CXCR4 is present in EC of pathologic microvessels. Moreover expression of CXCR4 appears to correlate with expression of IL-6, a cytokine with potent proinflammatory activity in context of inflammation. Further more, we show that a constant VEGF expression of leaflet surface ECs is associated with VEGFR1 expression of ECs within pathologic neovessels and that those neovessels are not exclusively confined to calcified areas with inflammatory infiltrate but—even if distributed in a sparse pattern—can be observed in noncalcified areas of diseased valves, as well as in nonsymptomatic valves lacking obvious calcification in light-microscopy, but revealing endothelium-adherent crystalline ultrastructures in SEM and IR spectrography.

The development of new pharmacologic therapies for this disease requires a clearer understanding of the mechanisms of disease onset. Angiogenic factors and proinflammatory cytokines are present and could represent an early witness of subsequent inflammatory activity, finally leading to fibrous remodeling and symptomatic valvulopathy.

## Figures and Tables

**Table 1:** Chemical composition of crystalline ultrastructures in inconspicuous valve areas (HNCA) and in massive calcifications of the same samples (HCA) given by FT-IR: proportions of proteines (Prot) and tendencially triclycerides (TGLs) are higher in areas with crystalline ultrastructures. Carbonated calcium hydroxylapatite (CA) is present in inconspicuous areas, while amorphous carbonated calcium phosphate (ACCP) is only present in massive calcifications. No significant differences were found for cholesterol (Chol) or whewellite (C1).

Samples	CA	ACCP	Prot	TGL	Chol	C1
HCA 1	59%	18%	20%	3%	—	—
HCA 2	47%	20%	20%	8%	5%	—
HCA 3	56%	20%	20%	4%	—	—
HCA 4	57%	15%	20%	5%	—	3%
HNCA 1	10%	—	87%	3%	—	—
HNCA 2	11%	—	75%	9%	5%	—
HNCA 3	12%	—	82%	6%	—	—
HNCA 4	5%	—	90%	2%	—	—
Control	12%	—	72%	12%	4%	—
